# Human Intestinal Epithelial Cells Release Antiviral Factors That Inhibit HIV Infection of Macrophages

**DOI:** 10.3389/fimmu.2018.00247

**Published:** 2018-02-19

**Authors:** Le Guo, Xi-Qiu Xu, Li Zhou, Run-Hong Zhou, Xu Wang, Jie-Liang Li, Jin-Biao Liu, Hang Liu, Biao Zhang, Wen-Zhe Ho

**Affiliations:** ^1^Wuhan University School of Basic Medical Sciences, Wuhan, China; ^2^Department of Pathology and Laboratory Medicine, Lewis Katz School of Medicine, Temple University, Philadelphia, PA, United States

**Keywords:** human intestinal epithelial cells, HIV, macrophages, toll-like receptor 3, interferons, IFN-stimulated genes, exosomes

## Abstract

As a rich source of CD4^+^ T cells and macrophages, the gastrointestinal (GI) tract is a major target site for HIV infection. The interplay between GI-resident macrophages and intestinal epithelial cells (IECs) constitutes an important element of GI innate immunity against pathogens. In this study, we investigated whether human IECs have the ability to produce antiviral factors that can inhibit HIV infection of macrophages. We demonstrated that IECs possess functional toll-like receptor 3 (TLR3), the activation of which resulted in induction of key interferon (IFN) regulatory factors (IRF3 and IRF7), IFN-β, IFN-λ, and CC chemokines (MIP-1α, MIP-1β, RANTES), the ligands of HIV entry co-receptor CCR5. In addition, TLR3-activated IECs release exosomes that contained the anti-HIV factors, including IFN-stimulated genes (ISGs: ISG15, ISG56, MxB, OAS-1, GBP5, and Viperin) and HIV restriction miRNAs (miRNA-17, miRNA-20, miRNA-28, miRNA-29 family members, and miRNA-125b). Importantly, treatment of macrophages with supernatant (SN) from the activated IEC cultures inhibited HIV replication. Further studies showed that IEC SN could also induce the expression of antiviral ISGs and cellular HIV restriction factors (Tetherin and APOBEC3G/3F) in HIV-infected macrophages. These findings indicated that IECs might act as an important element in GI innate immunity against HIV infection/replication.

## Introduction

The gastrointestinal (GI) tract has the largest mucosal surface in the body and serves as an important barrier between pathogens in the external environment and the body’s sterile internal environment (1). Tight epithelial junctions together with the GI immune system protect the host from pathogenic invasion. The GI tract is rich in HIV target cells, mainly activated CD4^+^ T cells and macrophages. Therefore, the GI tract is a major site for HIV infection. As first layer cells in the GI tract, intestinal epithelial cells (IECs) constantly exposed to HIV or HIV-infected cells, which could have a profound impact on the immune and barrier functions of the GI tract (2). In addition, IECs express galactosylceramide and HIV co-receptor CCR5 (3), which facilitate translocation of CCR5-tropic HIV from the apical to the basolateral surface *via* vesicular transcytosis (4, 5).

Central to the capacity of IECs to maintain barrier and immunoregulatory functions is their ability to act as frontline sensors to their microbial encounters and to integrate commensal bacteria-derived signals into antimicrobial and immunoregulatory responses (6). Studies have shown that the IECs express pattern-recognition receptors (PRRs) that enable them to act as dynamic sensors of the microbial environment and as active participants in directing mucosal immune cell responses (7). Among PRRs, toll-like receptor 3 (TLR3) in conjunction with TLR7 and TLR9 constitutes an effective system to monitor viral infection and replication. TLR3 is known to recognize viral double-stranded RNA (dsRNA), while TLR7 and TLR9 detect single-stranded RNA (ssRNA) and cytosine phosphate guanine DNA, respectively (8). Therefore, expressing functional TLR3, 7 and 9 in IECs play a crucial role in virus-mediated GI innate immune responses (9).

Macrophages present in the GI system constitute a major cellular reservoir for HIV due to the abundance of these cells at mucosal sites. GI-resident macrophages represent the largest population of mononuclear phagocytes in the body (10). In the rectum, there are more than three times as many CD68^+^ macrophages expressing CCR5 as those in the colon (4). The high expression of CCR5 on rectal macrophages suggests that the most distal sections of the gut may be especially vulnerable to HIV infection. Macrophages constitute up to 10% of infected cells in HIV-infected individuals (11, 12). HIV-Infected macrophages can transfer virus with high-multiplicity to CD4^+^ T cells and reduce the viral sensitivity to antiretroviral therapy and neutralizing antibodies (13, 14). In mucosa infiltrating, macrophages also play a role in systemic HIV spread (5). Macrophage activation contributes to HIV-mediated inflammation, as they can produce and release inflammatory cytokines that induce systemic immune activation, a hall marker of HIV disease progression. Conversely, macrophages play an important role in the host defense against HIV infection. Macrophages are a major producer of type I interferons (IFNs). Our early investigations (15, 16) showed that TLR3 activation of macrophages produced multiple intracellular HIV restriction factors and potently suppressed HIV infection/replication. However, the ability of macrophages to produce type I IFNs are significantly compromised by HIV infection. HIV blocks IFN induction in macrophages by inhibiting the function of a key kinase (TBK1) in the IFN signaling pathway through viral accessory proteins (Vpr and Vif) (17). In addition, HIV infection downregulates the antiviral IFN-stimulated genes (ISGs) (ISG15, OAS-1, and IFI44) in primary macrophages (18).

Exosomes play a key role in intercellular communication and innate immune regulation. A recent study showed that exosomes are formed in an endocytic compartment of multi-vesicular bodies (19). Exosomes are involved in many biological processes such as tissue injury and immune responses by transfer of antigens, antigen presentation (20), and the shuttling of proteins, mRNAs, and miRNA between cells (21). As such, it has been postulated that exosomes mediate intercellular communication by delivering functional factors to recipient cells (22). IEC lines also can secrete exosomes bearing accessory molecules that constitute a link between luminal antigens and local immune system (23). Studies have documented that the bystander cells can produce and release the exosomes, which contain multiple antiviral factors that can inhibit viral replication in target cells, including hepatitis B virus (24), HCV (25), and HIV (26, 27).

Evidently, the interplay between GI-resident macrophages and IECs has a key role in the GI innate immunity against viral infections. Unlike macrophages, IECs are not a host for HIV infection/replication, and it is unlikely that HIV has a direct and negative impact on functions of IECs. However, because IECs in the GI tract have to encounter a number of stimuli and immune cells, including HIV-infected macrophages (28), the activation of these non-immune cells in the GI tract is inevitable. Recent studies (19, 29) have shown that IECs can be induced to express and secrete specific arrays of cytokines, chemokines, and antimicrobial defense molecules, which is crucial for activating intestinal mucosal innate and adaptive immune responses. However, there is little information about whether the IECs are involved in the GI innate immunity against HIV infection. Specifically, it is unknown whether the IECs possess functional TLRs that can be immunologically activated to produce anti-HIV factors. Therefore, this study aimed to determine whether IECs have the ability to mount TLR3-IFN-mediated antiviral activities against HIV infection of macrophages.

## Materials and Methods

### Reagents

All culture plastic ware were obtained from Corning (Corning, NY, USA). Lyovec transfection reagent and Polyinosinic-polycytidylic acid (Poly I:C) (TLR3 ligand), Imiquimod (TLR7 ligand), ssRNA40 (TLR8 ligand), ODN2006 (TLR9 ligand) were purchased from InvivoGen (San Diego, CA, USA). All culture reagents were purchased from Gibco (Grand Island, NY, USA). Exosome-depleted fetal bovine serum (FBS) was purchased from System Biosciences, Inc. (Mountain View, CA, USA).

### Cell Culture

The human intestinal epithelial cell line (NCM460), originally derived from the normal colonic mucosa of a 68-year-old Hispanic male, were expanded in RPMI-1640 medium (30). Cells were cultured at 37°C with 5% CO_2_ and 100% humidity, and culture medium was changed every 3 days. To polarize IECs, we used a transwell system (31, 32), in which IECs (1 × 10^5^ cells/well) were grown on a 0.4 µm pore sized, 6.5 mm diameter transwell insert. The transepithelial electrical resistance was measured by Ohm meter. The cell cultures were considered to constitute a polarized epithelial monolayer when resistances were ≥600 Ω × cm^2^ and stable (33). Purified human peripheral blood monocytes were purchased from Human Immunology Core at the University of Pennsylvania (Philadelphia, PA, USA). The Core has the Institutional Review Board approval for blood collection from healthy donors. Freshly isolated monocytes were cultured in the 48-well plate (2.5 × 10^5^ cells/well) in DMEM containing 10% FBS. Macrophages refer to 7-day cultured monocytes.

### TLRs Activation

Lyovec was used for the transfection of the TLR ligands. IECs seeded on 48-well plates (5 × 10^4^/well) were transfected with Poly I:C (10 µg/ml), Imiquimod (10 µg/ml), ssRNA40 (10 µg/ml), ODN2006 (5 µM). Lyovec-treated cells were used as a vehicle control.

### Exosome Isolation

Intestinal epithelial cells were transfected with poly I:C (0.1, 1, 10 µg/ml) for 4 h and fresh-culturing medium containing 10% exosome-free FBS was added. At 48 h post-transfection, IECs supernatant (SN) was collected and exosomes were isolated through multiple rounds of centrifugation and filtration as previously reported (24). Briefly, 10 ml of SN were centrifuged at 300 × *g* for 10 min to remove floating cells, then at 2,000 × *g* for 10 min, and 10,000 × *g* for 30 min to remove cell debris, shedding vesicles, and apoptotic bodies. Finally, exosomes pellet were collected by ultracentrifugation at 100,000 × *g* for 70 min. For further purification, the pellets were washed with phosphate buffered saline (1× PBS) (Gibco, NY, USA) and centrifuged at 100,000 × *g* for 70 min. The pellet was resuspended in 100 μl 1× PBS, then immediately stored at −80°C until use.

### Immunofluorescence of Exosome

Macrophages were cultured at a density of 2.0 × 10^5^ cells/well in 48-well plates. Isolated exosomes from IECs SN were labeled with PKH67 Fluorescent according to the manufacturer’s protocol (Sigma-Aldrich). Purified PKH67 exosomes were incubated with macrophages and cultured at 37°C for 18 h in a CO_2_ incubator. Macrophages were then stained with a PKH26 Fluorescent for membrane and Hoechst 33342 for nuclei and washed three times with 1× PBS. The cells were photographed under a confocal microscope (Nikon A1R, Nikon, Japan).

### qRT-PCR Quantification of mRNA and miRNA

Total RNA from cultured cells was extracted with Tri-Reagent (Molecular Research Center, OH, USA) as previously described (34). Total RNA (1 µg) was subjected to reverse transcription (RT) using reagents from Promega (Promega, WI, USA). The RT system with random primers for 1 h at 42°C. The reaction was terminated by incubating the reaction mixture at 99°C for 5 min, and the mixture was then kept at 4°C. The resulting cDNA was then used as a template for qPCR quantification. The qPCR was performed with iQ SYBR Green Supermix (Bio-Rad Laboratories, CA, USA) as previously described (35). Thermal cycling conditions were designed as follows: initial denaturation at 95°C for 3 min, followed by 40 cycles of 95°C for 10 s, and 60°C for 1 min. miRNA was extracted from IECs-derived exosomes using the miRNeasy Mini Kit (Qiagen, CA, USA) in accordance with the manufacturer’s instruction and reverse-transcribed with a miScript Reverse Transcription Kit (Qiagen, CA, USA). qRT-PCR was carried out using miScript Primer Assays and miScript SYBR Green PCR Kit from Qiagen as previously described (36). Synthetic caenorhabditis elegans miRNA-39 (cel-miR-39) was used as a spiked-in miRNA for normalization.

### Western Blot

Total cell lysates of IECs transfected with Poly I:C was prepared by using the cell extraction buffer (Thermo Fisher Scientific, MA, USA) according to the manufacturer’s instructions. Equal amounts of protein lysates (30 µg) were separated on 4–12% sodium dodecyl sulfate polyacrylamide gel electrophoresis precast gels and transfected to an Immunobiolon-P membrane (Millipore, Eschborn, Germany). The blots were incubated with primary antibodies in 5% nonfat milk in PBS with 0.05% Tween 20 (PBST) overnight at 4°C (IRF3, 1:1,000; Phospho-IRF3, 1:1,000; IRF7, 1:1,000; Phospho-IRF7, 1:1,000; GAPDH, 1:5,000; β-actin, 1:5,000; EEA1, 1:1,000; CD63, 1:1,000; LAMP2, 1:2,000; Alix, 1:1,000; ISG15, 1:1,000; ISG56, 1:1,000; GBP5, 1:1,000; Viperin, 1:1,000; MxA, 1:1,000; MxB, 1:1,000; OAS-1, 1:1,000). All antibodies were obtained from Cell Signaling Technology (Cell Signaling Technology, MA, USA) Horseradish peroxidase-conjugated appropriate second antibodies were diluted at 1:2,000 to 1:8,000 in 2% nonfat milk PBST. Blots were developed with SuperSignal West Pico Chemiluminescent Substrate (Thermo Fisher Scientific, MA, USA).

### ELISA

Interferon-β and IFN-λ protein levels in IECs culture SN were measured with ELISA (IFN-β: Invitrogen; IFN-λ1/3, IFN-λ2: R&D system Inc., MH, USA). Assays were carried out according to the manufacturer’s instructions.

### Cytometric Bead Array (CBA) Assay

The CBA assay was performed to simultaneously measure CC chemokines (MIP1-α, MIP1-β, and RANTES) levels in cell culture supernatant, according to the instructions of the manufacturer (BD Biosciences, CA, USA).

### Macrophage Treatment and HIV Infection

Macrophages were pretreated for 24 h with SN (10%, v/v) or exosomes (2 µg/ml, equal to the amount of 10% SN) from IECs cultures collected at 48 h post-stimulation with Poly I:C. HIV Bal strain was obtained from the AIDS Research and Reference Reagent Program at the National Institution of Health (NIH). Macrophages were incubated with cell-free HIV Bal (p24, 20 ng/ml) overnight, and cells were then washed three times with fresh DMEM. During the postinfection period, SN or exosomes were added to the macrophages where appropriate. At day 8 postinfection, cell and SN samples were collected for HIV GAG gene expression. To determine whether the polarized stimulation of IECs could mediate HIV inhibition in macrophages. Poly I:C (1 µg/ml) was added to the upper or lower chamber of IECs cultures. Culture SN was collected 48 h after Poly I:C transfection. Cell-associated HIV GAG gene expression in macrophages treated with 10% [volume to volume ratio (v/v)] of indicated SN was measured by qRT-PCR at 96 h post-infection. To deplete exosomes, the SN from Poly I:C-stimulated IECs were incubated with anti-CD63 antibody-conjugated Dynabeads overnight at 4°C and then separated in a magnetic field. For detection of early products of Strong-Stop DNA in macrophages, SN from TLR3-activated IECs cultures was added to macrophages cultures 24 h prior to infection with DNase I-treated HIV Bal for 3 h. Cellular DNA, including genomic and viral DNA products, was then isolated with the Tri-Reagent. Strong-stop DNA, the first product of HIV RT, was analyzed by the qPCR with primers specific for strong-stop DNA. The DNA concentrations of the each sample were normalized by equal DNA loading confirmed with primers for GAPDH.

### Data Analysis

Data were presented as the mean ± SD from at least three independent experiments, and statistical significance was measured by Student’s *t*-test or one-way analysis of variance followed by the Newman–Keul’s test where appropriate. Statistical significance was defined as *P* < 0.05 or *P* < 0.01.

## Results

### TLR3 Signaling of IECs Induces IFNs

Activation of TLRs 3, 7, and 9 could trigger intracellular IFN-mediated innate immunity against virus infections (37). Therefore, we first examined the expression of TLRs in IECs. As shown in Figure S1A in Supplementary Material, IECs expressed mRNAs for all known human TLRs except TLR5. To investigate whether the antiviral TLRs (TLR3, 7, 9) are biologically functional in IECs, we transfected the cells with the ligands to TLR3 (Poly I:C), TLR7 (Imiquimod), TLR8 (ssRNA40), and TLR9 (ODN2006). As shown in Figure S1B in Supplementary Material, the IECs expressed functional TLR3 and TLR8, as the ligands to these TLRs could induce the expression IFN-β and IFN-λ. In contrast, the ligands of TLR7 and TLR9 had little effect on IFN induction. TCI, a TLR3 complex inhibitor, could significantly block the effect of Poly I:C (Figure S2 in Supplementary Material). We thus focused on the impact of TLR3 signaling on IFN induction in IECs in the subsequent experiments.

**Figure 1:**
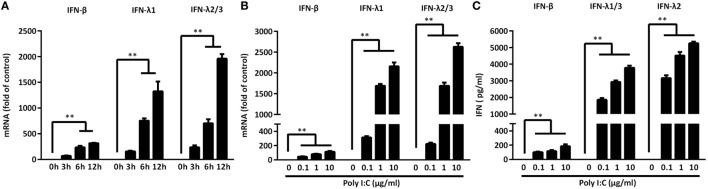
Toll-like receptor 3 signaling induces interferon (IFN)-β and IFN-λ expression. **(A)** Intestinal epithelial cells (IECs) were transfected with Poly I:C (1 µg/ml) for the indicated times. Dose-dependent effect of Poly I:C on IFN induction of IECs at **(B)** mRNA and **(C)** protein levels. Data shown were the mean ± SD of three independent experiments. Asterisks indicate that the differences between the indicated groups are statistically significant (**P < 0.01).

**Figure 2:**
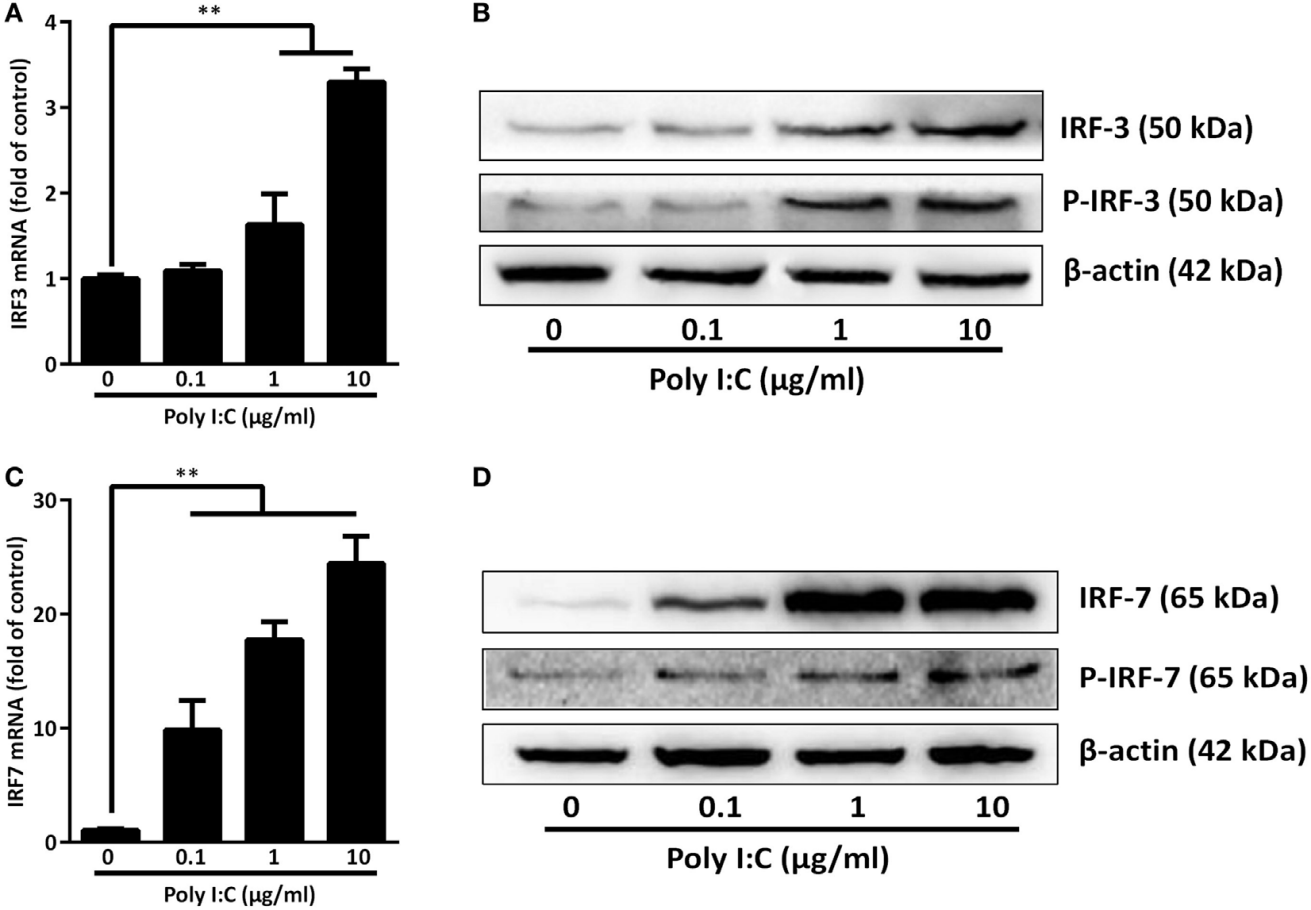
Effect of toll-like receptor 3 activation on IRFs. intestinal epithelial cells were transfected with or without Poly I:C at indicated concentrations for 3 or 6 h. **(A,C)** For mRNA quantification, total cellular RNA was collected at 3 h post-transfection and subjected to the qRT-PCR. **(B,D)** For protein quantification, cellular proteins were collected at 6 h post-transfection and subjected to immunoblot. β-actin serves as the loading control. P-IRF3:Phospho-IRF3; P-IRF7:Phospho-IRF7. Data shown were the mean ± SD of three independent experiments. Asterisks indicate that the differences are statistically significant (**P < 0.01).

As shown in Figure 1, TLR3 activation of IECs induced IFN-β and IFN-λ at both mRNA (Figure 1B) and protein (Figure 1C) levels. These effects of Poly I:C stimulation on IFN-β and IFN-λ expression in IECs were dose- and time-dependent (Figures 1A,B). We next examined whether IRF3 and IRF7, key regulators of the IFN signaling pathway, are involved in the TLR3 action on IFN induction by IECs. As shown in Figure 2, TLR3 signaling of IECs induced the phosphorylation of both IRF3 and IRF7, which were positively associated with the dose of Poly I:C transfected into IECs.

### IECs-Derived Exosomes Can Be Taken up by Macrophages

**Figure 3:**
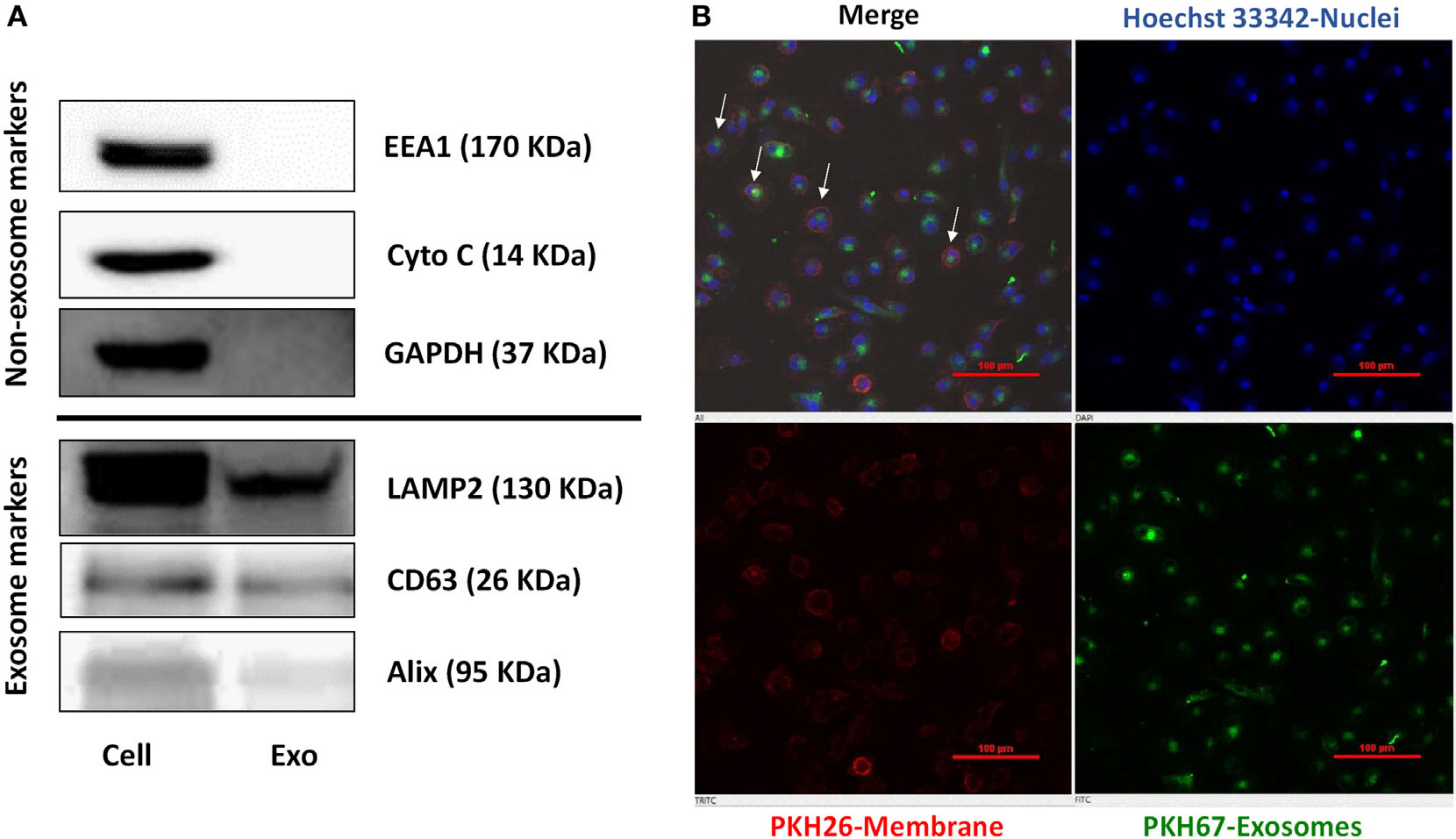
Characterization of exosomes and delivery of intestinal epithelial cells (IECs) exosomes (Exo) to macrophages. **(A)** The expression of exosomal markers (Lamp2, Alix, CD63), and nonexosomal markers (EEA1, Cytochrome C, GAPDH) was determined by immunoblot. **(B)** The uptake of IECs exosomes labeled with PKH67 by macrophages. Macrophages were incubated with PHK67-labeled IECs exosomes (green) for 24 h and then stained with PKH26 for general cell membrane labeling (red) and Hoechst 33342 (blue) for nuclei. Data are representative of three independent experiments. Scale bar, 100 **µ**m.

Exosomes released from donor cells could deliver their cargo to recipient cells and subsequently modulate host cell function (21). We thus isolated and characterized the exosomes from activated IECs cultures by detecting the common exosome-carried proteins (Alix, CD63, and LAMP2) (Figure 3A). To determine whether macrophages (recipient cells) can take up the exosomes released from IECs, we incubated macrophages with exosomes labeled with green fluorescent dye PKH67. As shown in Figure 3B, PKH67-labeled exosomes were observed within macrophages treated with SN from activated IECs cultures.

### IECs-Isolated Exosomes Carry the Antiviral ISGs and miRNAs

**Figure 4:**
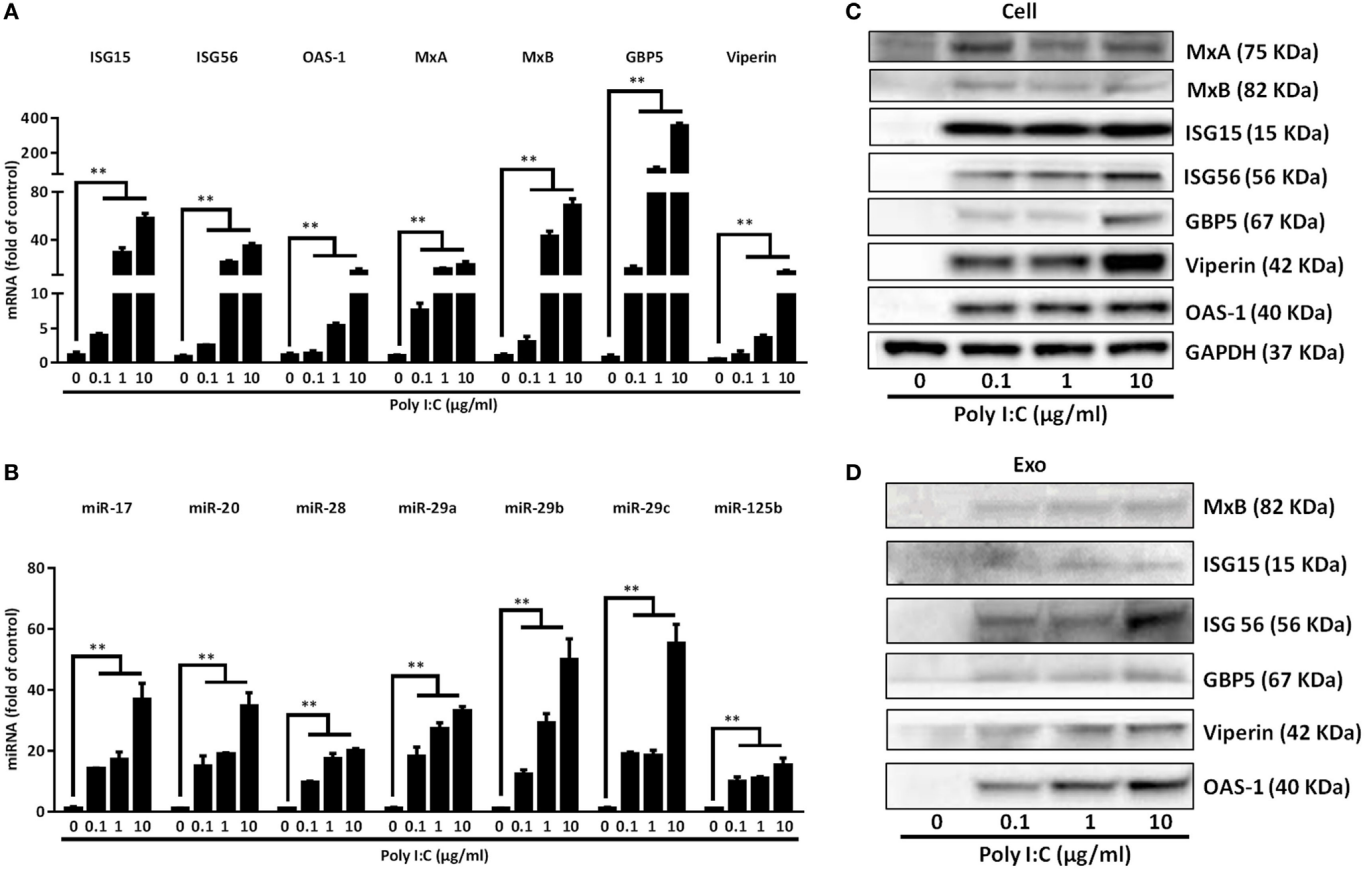
Characterization of the antiviral factors in the cells and exosomes of toll-like receptor 3 signaling of intestinal epithelial cells (IECs). IECs were transfected with or without Poly I:C at indicated concentrations. **(A)** For IFN-stimulated genes (ISGs), mRNA quantification, total cellular RNA was collected at 12 h post-transfection and subjected to the qRT-PCR. **(B)** IECs cultured in exosome-free media were transfected with or without poly I:C (1 µg/ml) for 48 h. miRNA in secreted exosomes from IECs supernatant were quantified by qRT-PCR. Synthetic caenorhabditis elegans miRNA-39 (cel-miR-39) was used as a spiked-in miRNA for normalization. Levels of miRNAs were plotted as fold of control. **(C)** For protein quantification, cellular proteins were collected at 24 h post-transfection and subjected to immunoblot. GAPDH serves as the loading control. **(D)** Exosomal protein was collected at 48 h and subjected to immunoblot with indicated ISGs antibodies. 20 μg of total exosome loaded. Data shown represent the mean ± SD of three independent experiments. Asterisks indicate statistically significant differences. (**P < 0.01).

Next, we investigated whether the exosomes from activated IECs contained the antiviral ISGs and miRNA. As shown in Figures 4A,C, TLR3 signaling of IECs induced the expression of ISG15, ISG56, OAS-1, MxA, MxB, GBP5, and Viperin at both mRNA and protein levels. In addition, there were elevated levels of these ISGs in the exosomes isolated from Poly I:C-stimulated IECs (Figure 4D). We also found that the anti-HIV miRNAs: miRNA-17, miRNA-20, miRNA-28, miRNA-29 family members (miR-29a, 29b, and 29c) and miRNA-125b were increased in the exosomes (Figure 4B) from Poly I:C-stimulated IECs.

### TLR3 Signaling of IECs Inhibits HIV Infection of Macrophages

**Figure 5:**
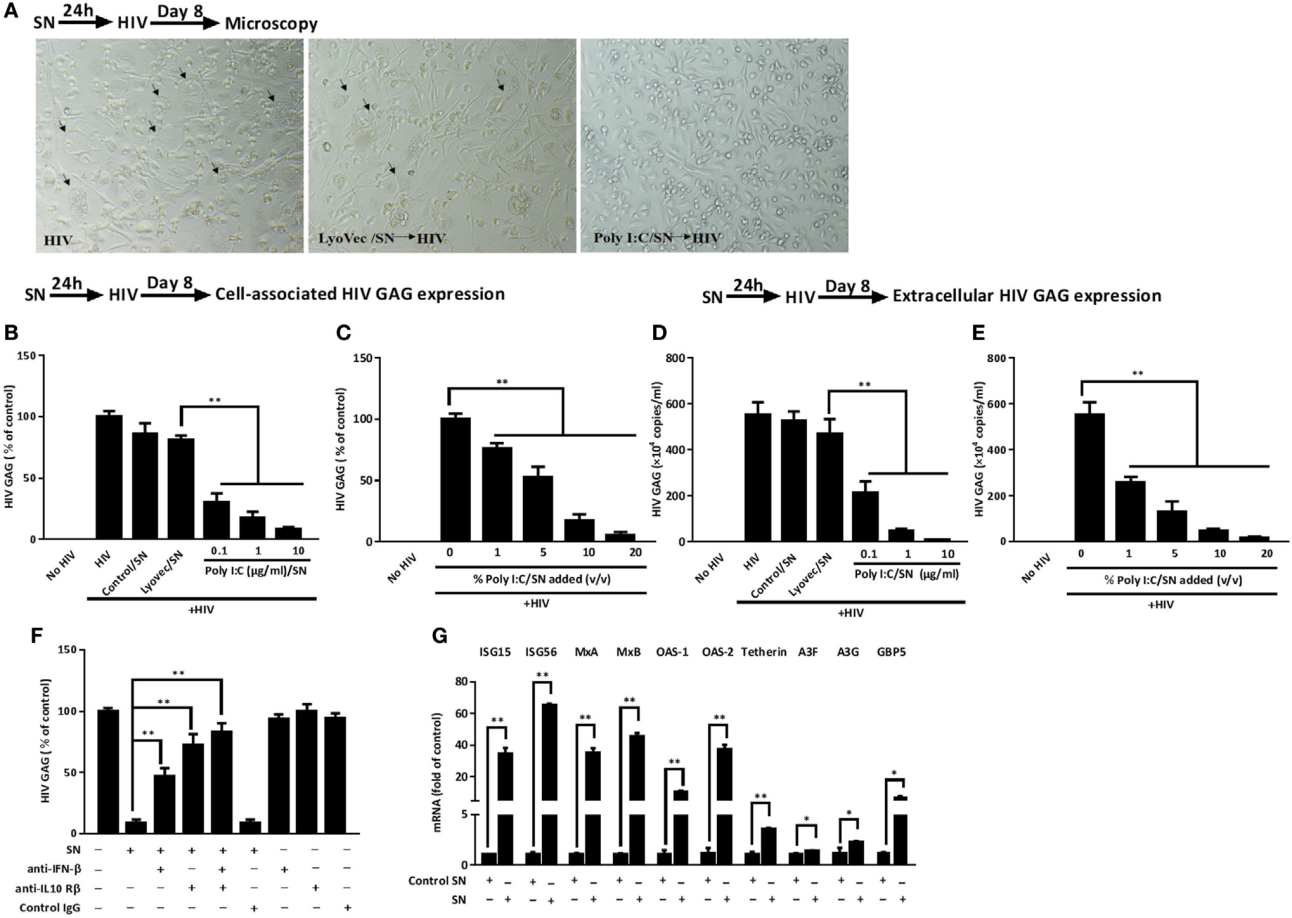
Effect of supernatant (SN) from intestinal epithelial cells (IECs) cultures inhibits HIV replication in macrophages. **(A)** Morphologic observations of HIV-infected macrophages with mock treatment or pretreated with either LyoVec/SN or Poly I:C/SN (arrows indicate syncytium, magnification × 100). **(B,C)** Cell-associated and **(D,E)** extracellular HIV GAG gene expression level in macrophages with 10% [volume to volume ratio (v/v)] of indicated SN pretreatments or with indicated volumes of 1 µg/ml Poly I:C-stimulated IECs SN pretreatments was measured by qRT-PCR at 8 days postinfection. **(F,G)** Roles of interferon (IFN)-β and IFN-λ in IECs SN-mediated anti-HIV activity and the effect of IECs SN on the expression of IFN-stimulated genes (ISGs) in macrophages. **(F)** Effect of neutralization antibodies (Abs) to IFN-β or IFN-λ receptor on IECs culture SN-mediated anti-HIV activity. IECs SN was preincubated with anti-IFN-β (10 µg/ml) for 1 h and then used to treat macrophages 24 h prior to HIV Bal infection (p24, 20 ng/ml). For IFN-λ receptor pretreatment, the anti-IL10Rβ neutralization antibody (10 µg/ml) was added to macrophage cultures for 1 h prior to the addition of SN. HIV GAG expression was then measured by qRT-PCR for 8 days postinfection. **(G)** Effect of Poly I:C-stimulated IECs culture SN on ISG expression of macrophages. IECs were stimulated with Poly I:C for 48 h and culture SN was collected for treatment of macrophages (10% v/v) for 12 h. RNA was extracted, and the expression of ISGs was measured by qRT-PCR. Representative data were the mean ± SD of three independent experiments using macrophages of three donors. Asterisks indicate that the differences between the indicated groups are statistically significant (*P < 0.05, **P < 0.01).

As shown in Figure 5A, macrophages treated with SN from Poly I:C-stimulated IECs cultures had less HIV infection-induced syncytia than untreated cells. In addition, HIV GAG gene expression was suppressed in macrophages pretreated with SN from Poly I:C-stimulated IECs cultures (Figures 5B–E). This IECs SN-mediated HIV inhibition was positively associated with the concentrations of Poly I:C used to activate IECs (Figures 5B,D) and the percentage of IECs SN added to macrophage cultures (Figures 5C,E). To decipher the roles of each subtype of IFNs in IECs-mediated anti-HIV activity, we used the neutralization antibody against IFN-β to pretreat the IECs SN or antibody against IFN-λ receptor to pretreat macrophages, respectively. As shown in Figure 5F, antibody to IFN-β significantly reduced the anti-HIV activity of SN from activated IECs cultures. In addition, anti-IL10 receptor β (IL-10Rβ a subunit of IFN-λ receptor) antibody pretreatment of macrophages could also block the anti-HIV activity of the IECs SN. We then investigated whether ISGs could be induced in macrophages treated by IECs SN. As shown in Figure 5F, TLR3 signaling of IECs induced the expression of ISG (ISG15, ISG56, OAS-1, OAS-2, MxA, MxB, GBP5, and Viperin) and several known HIV restriction factors, including Tetherin and APOBEC3G/3F in macrophages.

**Figure 6:**
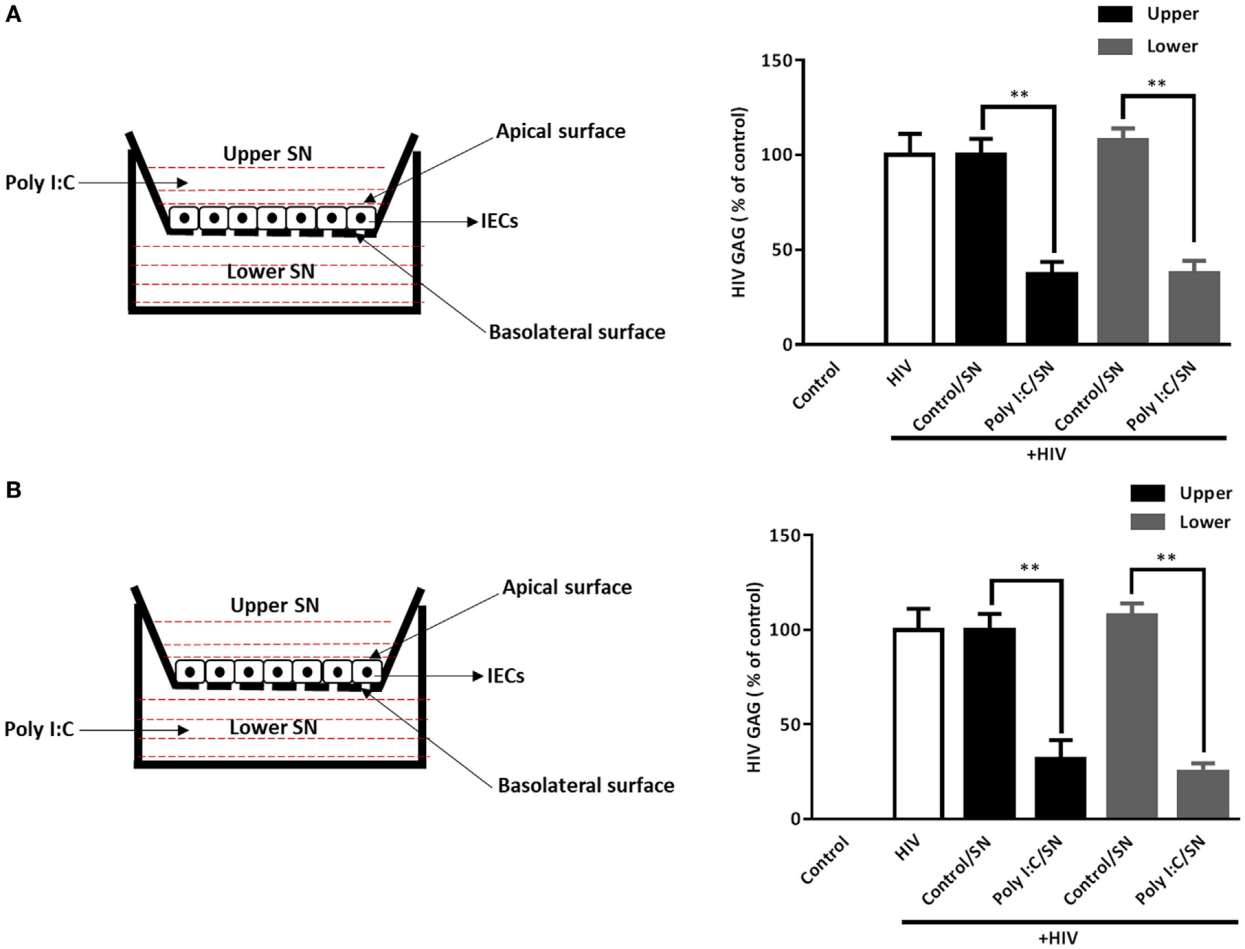
Supernatant (SN) of polarized intestinal epithelial cells (IECs) cultures inhibits HIV replication in macrophages. IECs were seeded onto a transwell insert at a density of 1 × 10^5^ cells/insert and cultured for 72 h prior to use. The integrity of the IEC monolayer in each well was assessed for the development of transepithelial electrical resistance (TEER). Poly I:C (1 µg/ml) was then added to the upper [**(A)**, apical level] or lower [**(B)**, basolateral level] chamber of the IECs cultures. The SN was collected 48 h after Poly I:C treatment. Cell-associated HIV GAG gene expression in macrophages treated with 10% [volume to volume ratio (v/v)] of indicated SN was measured by qRT-PCR at 96 h postinfection. Data shown were the mean ± SD of three independent experiments. Asterisks indicate statistically significant differences (**P < 0.01).

To ensure the IECs cultures are polarized (38), we used the transwell system to determine whether the polarized stimulation IECs could mediate HIV inhibition in macrophages. As shown in Figure 6, HIV GAG gene expression was suppressed in macrophages treated with SN from either upper (apical side) or lower (basolateral side) chambers of the transwell cultures. No significant difference in HIV inhibition was observed between SN from the upper level chambers and those from the lower level chambers.

### IECs-Derived Exosomes Contribute to HIV Inhibition in Macrophages

**Figure 7:**
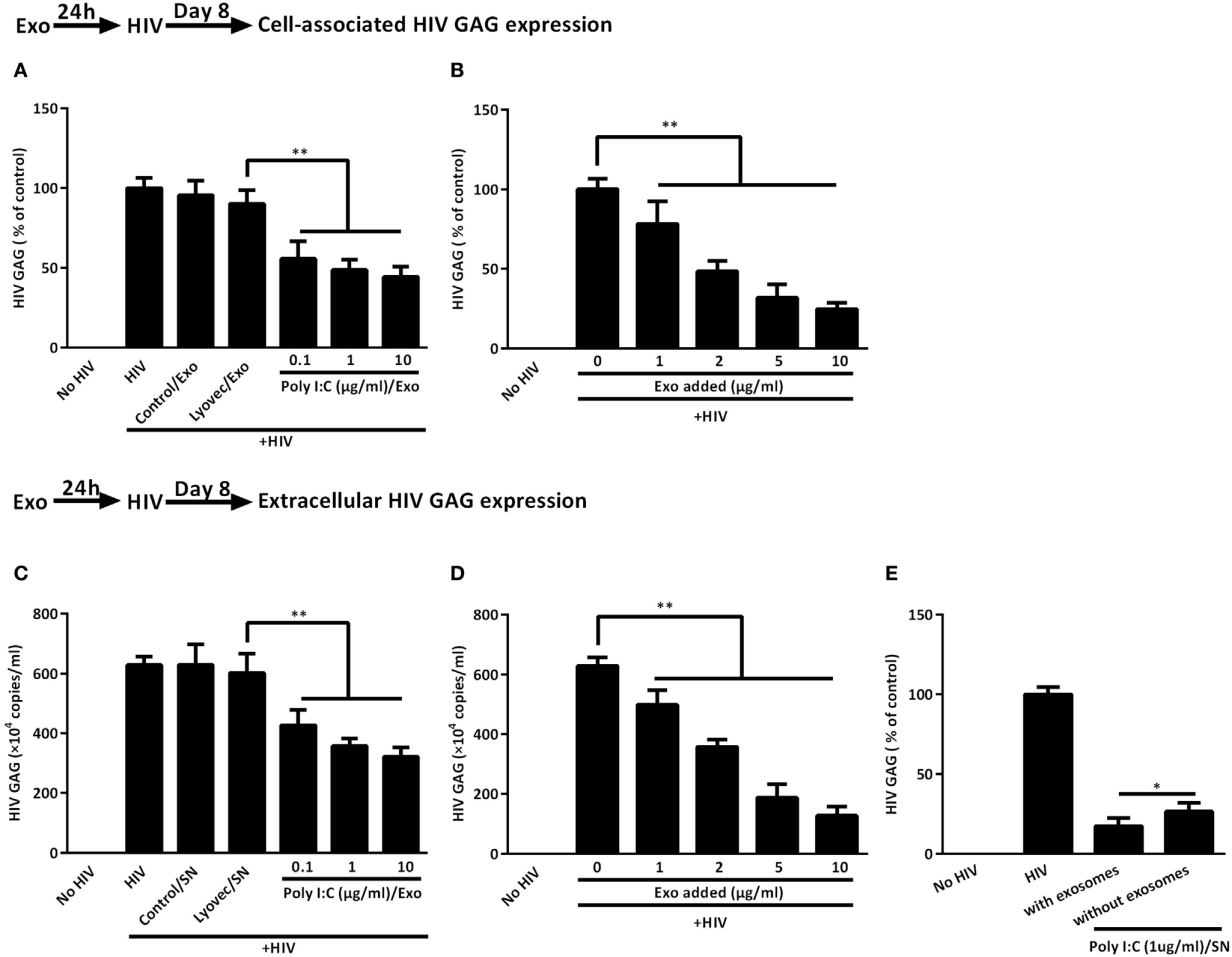
Intestinal epithelial cells (IECs)-derived exosomes contribute to IECs supernatant (SN)-mediated HIV inhibition in macrophages. **(A,B)** Cell-associated and **(C,D)** extracellular HIV GAG gene expression in macrophages with 2 µg/ml of indicated exosomes pretreatments or with the indicated concentration of 1 µg/ml Poly I:C-stimulated IECs exosomes pretreatments were measured by qRT-PCR for 8 days postinfection, respectively. **(E)** The inhibition of HIV replication by IECs culture SN with or without exosome depletion. To deplete exosomes, the SN from Poly I:C-stimulated IECs were incubated with anti-CD63 antibody-conjugated Dynabeads overnight at 4°C and then separated in a magnetic field. Representative data were the mean ± SD of three independent experiments using macrophages of three donors. Asterisks indicate that the differences between the indicated groups are statistically significant (*P < 0.05, **P < 0.01).

To evaluate the role of the exosomes in IECs-mediated anti-HIV activity in macrophages, we added the activated IECs-derived exosomes to macrophage cultures. As shown in Figures 7A–D, macrophages treated with the exosomes showed less expression of cell-associated as well as extracellular HIV GAG gene as compared with untreated macrophages. We then examined the anti-HIV potency of IECs SN with or without exosome depletion. As indicated in Figure 7E, SN from Poly I:C-stimulated IECs significantly suppressed HIV, while the depletion of exosomes from IECs SN diminished IECs-mediated anti-HIV activity in macrophages.

### TLR3 Signaling of IECs Induces CC Chemokines

**Figure 8:**
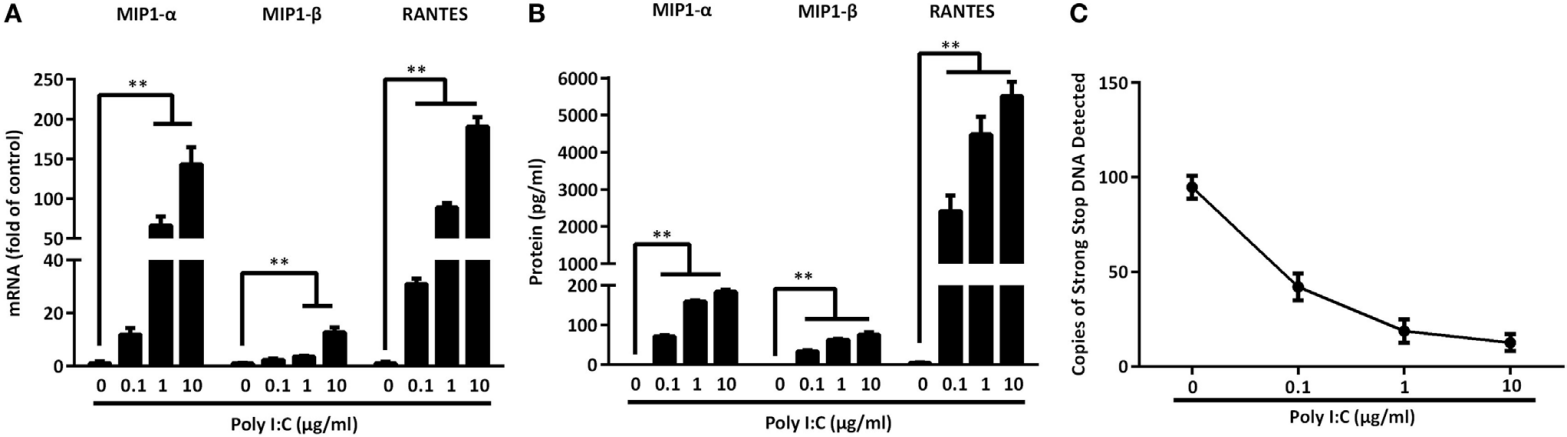
Toll-like receptor 3 signaling of intestinal epithelial cells (IECs) induces CC chemokines. IECs were transfected with or without Poly I:C at indicated concentrations for 12 h (mRNA) or 48 h (protein). **(A)** Cellular RNA was collected and subjected to the qRT-PCR. **(B)** MIP-1α, MIP-1β, and RANTES proteins were analyzed by Cytometric Bead Array with the specific kits according to the manufacturer’s instructions. **(C)** HIV strong-stop DNA was detected in macrophages with 10% (v/v) of supernatant from indicated doses of Poly I:C-treated IECs cultures. Representative data from at least three donor macrophages was shown. Asterisks indicate that the differences between the indicated groups are statistically significant (**P < 0.01).

CC chemokines (MIP-1α, MIP-1β, RANTES) are the ligands of the HIV entry co-receptor, CCR5. We examined whether IECs upon the TLR3 activation can produce these CC chemokines. As shown in Figure 8, Poly I:C treatment of IECs dose-dependently induced the CC chemokines at both mRNA (Figure 8A) and protein (Figure 8B) levels. We then examined the ability of IECs SN to block HIV entry into macrophages. As shown in Figure 8C, the pretreatment of macrophages with the IECs SN resulted in a marked decrease in strong-stop DNA of HIV.

## Discussion

HIV infection provides ample pathogen-associated molecular patterns that can be detected by a variety of PRRs of the innate immune system (39). Among the PRRs, TLR3 is implicated in sensing dsRNA structures during viral infections, including HIV (40). While it has been reported that intestinal epithelial cell lines Caco-2 and HT-29 express functional TLR3 (41), there is little information about TLR3 activation of IECs and its role in antiviral activity against HIV infections of macrophages. We demonstrated that human IECs expressed functional TLR3, the activation of which resulted in the production of multiple antiviral factors, including the type I and III IFNs (Figure 1), ISGs, HIV restriction miRNAs (Figure 4), and CC chemokines (Figure 8). Importantly, we found that when added to primary human macrophage cultures, SN from the activated IECs cultures could potently suppress HIV infection and replication. In our early work of studying factors that influence the activation efficiency of TLR3 by Poly I:C (15), we found that the direct addition of Poly I:C to the cultures of primary macrophages or a neuroplastoma cell line could effectively activate TLR3. However, the transfection was necessary and needed in order to have efficient TLR3 activation by Poly I:C in the human hepatic cell line (Huh7) and brain microvascular endothelial cell line (hCMEC/D3). In addition, we demonstrated that the efficiency of TLR3 activation by high molecular mass Poly I:C was significantly higher than that by low molecular mass Poly I:C. These findings indicated that cell types and the size of Poly I:C are the crucial factors in Poly I:C-mediated TLR3 activation. As demonstrated in Figure S3 in Supplementary Material, we examined difference in the TLR3 activation efficiency between the direct addition and transfection of Poly I:C in IECs, showing that the levels of induced IFNs were significantly higher in IECs transfected with Poly I:C as compared to direct Poly I:C treatment. Therefore, we used the transfection technique for Poly I:C stimulation of IECs in this study to conceptually prove that as non-immune cells in GI tract, IECs can produce antiviral factors that can be transported through exosomes to macrophages, inhibiting HIV replication. The HIV inhibition in macrophages was also seen in macrophages treated with SN from either apical side or basolateral side of the polarized/activated IEC cultures (Figure 6). It was reported that there were little differences in TLR3 expression at different sites or between non-inflamed and inflamed mucosae in tissues from ulcerative colitis patients (42). Also, the polarized IECs responded to the TLR ligands, including TLR3, secreting IL-8 into the basolateral chamber, either exclusively on basolateral stimulation, or on apical stimulation. In non-polarized IECs, as expected, there was no difference in the response to all of these ligands (33).

Although IECs are non-immune cells, they are able to produce IFN-driven antiviral factors, including ISGs. Studies have shown that the ISGs, including ISG15, ISG56, MxA, MxB, OAS-1, OAS-2, and GBP5 have anti-HIV activities (43–45). ISG15 plays a crucial role in the IFN-mediated inhibition of late stages of HIV assembly and release (46); MxB inhibits HIV infection by inhibiting the capsid-dependent nuclear import of subviral complexes (47); GBP5 reduces HIV infectivity by interfering with Env processing and incorporation (48). In addition to the ISGs, Poly I:C-stimulated IECs expressed HIV restriction miRNAs (Figure 4), including miRNA-17, miRNA-20, miRNA-28, miRNA-29 family members (miR-29a, 29b, and 29c), and miRNA-125b. It is known that miRNA-28 and miRNA-125b can target the 3’UTR of HIV transcripts (49). miRNA-29 family members interfere with virus replication, as they can target a highly conserved site in various HIV subtypes (50). Studies have shown that miRNA-17 and miRNA-20 target p300/CBP associated factor (PCAF), a cellular cofactor of the HIV Tat protein (51). Furthermore, we found that CC chemokines (MIP-1α, MIP-1β, RANTES), ligands of HIV entry co-receptor CCR5, were induced in activated IECs (Figure 8). The observation evidenced the role of CC chemokines in IECs-mediated HIV inhibition that SN from TLR3-activated IEC cultures could block HIV entry into macrophages. IFN-β and IFN-λ in IECs SN appeared to be responsible for the induction of these anti-HIV factors, as the antibodies to IFN-β and IFN-λ receptors could block the inhibitory effect of IECs SN on HIV (Figure 5).

The investigation on the mechanisms for the induction of IFNs showed that there was upregulation of IRF3 and IRF7 in activated IECs (Figure 2). IRF3 and IRF7 are the key regulators of type I and III IFNs during viral infections (52). IRF3 and IRF7 phosphorylation is a crucial step in activating type I and III IFNs-mediated antiviral response (53). Both IRF3 and IRF7 require phosphorylation-induced activation in order to translocate to the nucleus to activate IFNs (54). Specifically, during viral infections, IRF3 is important in the early phase of inducing the transcription of IFN-α and IFN-β, which then can activate IRF7. Similar to IFN-β, IFN-λ1 gene is regulated by virus-activated IRF3 and IRF7, whereas IFN-λ2/3 gene expression is mainly controlled by IRF7 (55). IRF7 not only induces IFNs, but also actives many ISGs, among which PKR, OAS, and the Mx protein have been well characterized for their antiviral activities (56).

As one of the primary targets for HIV infection and persistence, macrophages have been implicated as an important HIV reservoir. Our early investigations (26, 57) showed that TLR3 activation of macrophages potently suppressed HIV infection and replication through multiple antiviral mechanisms at both the cellular and molecular levels. Despite being a major producer of type I IFNs, the biological functions of macrophages are significantly compromised in IFN induction upon HIV infection (17, 18). In contrast to macrophages, IECs are not the target of HIV. Therefore, it is unlikely that HIV has a direct and negative impact on IECs. As the first line of cells in the GI system, the IECs have to encounter a number of stimuli and immune cells, including HIV-infected macrophages (58). Thus, the activation of these nonimmune cells in the GI tract is inevitable. We found that activated IECs SN could induce the expression of several key HIV restriction factors in macrophages, including Tetherin and APOBEC3G/3F (Figure 5). Tetherin is a transmembrane protein that specifically inhibits HIV release from infected cells (59), APOBEC3G/3F are single-stranded DNA deaminases that inhibit HIV replication through deaminating cytidine to uracil on the minus strand of the HIV proviral DNA (60). Thus, the activation of IFN-mediated antiviral responses by IECs should be beneficial for GI protection. As a non-HIV target cell in the GI tract, it is unlikely that the ability of IECs to mount an IFN-mediated anti-HIV response would be compromised by HIV infection. We as well as others have shown that IFNs were produced not only by the immune cells but also by the nonimmune cells in the CNS, such as neurons and astrocytes (34, 61). In contrast to Poly I:C induction of both IFN-α and IFN-β in the immune cells, TLR3 signaling of IECs induced only IFN-β expression. This finding is consistent with the report by Starace et al. showing that Poly I:C induced IFN-β but not IFN-α in mouse Sertoli cells (62). These observations along with the findings of this study support the notion that IECs and other nonimmune cells in the GI tract could be important bystanders in mounting effective antiviral responses, which may have a key role in restricting HIV infection/replication in the GI system.

To understand how IECs could transport the antiviral factors to macrophages, we examined whether IECs can produce and release exosomes which are known to have the ability to shuttle biologically active molecules. Exosomes have a vital role in a variety of biologic processes, such as cell proliferation, apoptosis, and immune responses (63, 64). A major recent study in the intestinal mucosa field unveiled the capacity of exosomes to mediate the functional transfer of genetic materials (mRNAs and miRNAs) between immune cells (65). We found that IECs-derived exosomes could be taken up by infected macrophages, inhibiting HIV replication (Figure 7). We also observed that exosomes from Poly I:C-stimulated IECs were enriched with antiviral cellular ISGs and miRNAs (Figure 4), including miRNA-17, miRNA-20, miRNA-28, miRNA-29 family members (miR-29a, 29b, and 29c) and miRNA-125b. miRNA-28 and miRNA-125b are known to target 3’UTR of HIV transcripts (66). miRNA-29 family members interfere with virus replication, as they can target a highly conserved site in various HIV subtypes (50). Studies by several groups showed that miRNA-17 and miRNA-20 target p300/CBP associated factor (PCAF), a cellular cofactor of the HIV Tat protein (67).

**Figure 9:**
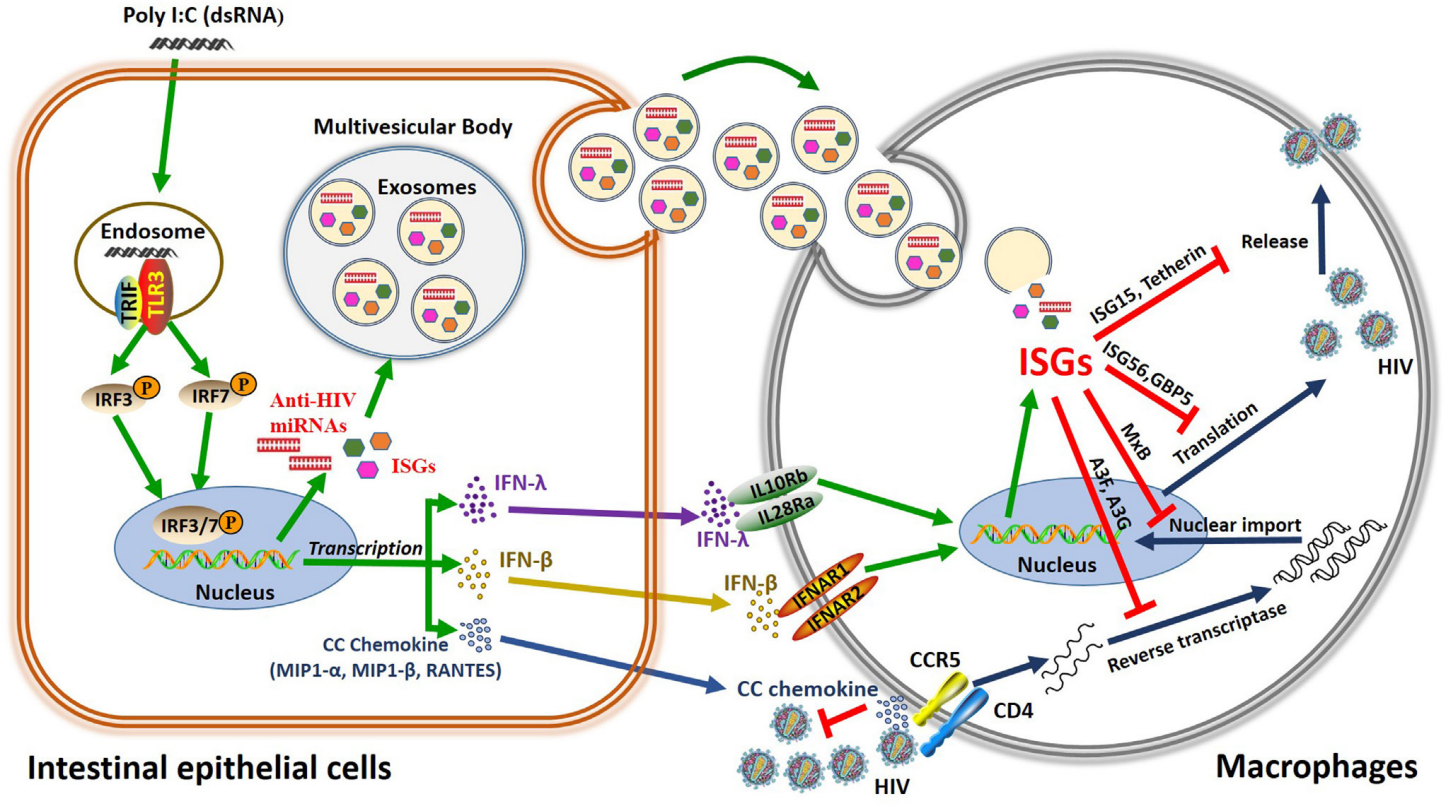
Schema of the anti-HIV mechanism of toll-like receptor 3 (TLR3) signaling of intestinal epithelial cells (IECs). Stimulation of IECs with double-stranded RNA (Poly I:C) activates TLR3 pathway, which facilitates phosphorylation and translocation of IRF3 and IRF7, initiating the transcription of interferon (IFN)-β, IFN-λ, and CC chemokine and releasing exosomes in the IECs. CC chemokines bind to HIV entry co-receptor CCR5 and block HIV entry. In addition, IFN-β and IFN-λ released from IECs can bind to their receptors in macrophages, inducing anti-HIV IFN-stimulated genes (ISGs) (ISG15, ISG56, MxA, MxB, OAS-1, OAS-2, GPB5, Tetherin, and APOBEC3G/3F), and exosome delivery of ISGs and miRNA to HIV-infected macrophages, which inhibit HIV at different steps of viral replication.

Collectively, we have provided the experimental evidence that TLR3 activation-induced antiviral factors in IECs could be transported to macrophages through exosomes released by IECs and internalized by macrophages (Figure 9). Because HIV has evolved several mechanisms to evade TLR3 mediated intracellular innate immunity in target cells, such as macrophages (68, 69), anti-HIV support from non-immune bystander cells is helpful in restoring the HIV-suppressed system in infected cells. Given that macrophage is an important cellular reservoir for HIV infection/persistence, to control and eradicate HIV in macrophages is clinically significant. Although the precise cellular and molecular mechanisms by which activated IECs could inhibit HIV replication in macrophages remain to be determined, the induction of IFNs, antiviral ISGs, HIV restriction miRNAs, and CC chemokines should account for much of IECs-mediated anti-HIV activity. However, further *in vitro* and *in vivo* investigations are necessary in order to determine whether the TLR3 signaling of IECs is indeed beneficial in protecting GI macrophages from HIV infection. Currently, the therapeutic TLR agonists are being developed for the treatment of cancer, allergies and viral infections. A number of TLR agonists are now in clinical or preclinical trails such as the anti-HIV TLR3 agonist (Poly I:C 12U) (70–72). These studies support the notion for further developing a TLR3 agonist-based therapy for HIV disease in which host cell innate immune responses are significantly compromised by the virus. These future studies are critical for the design and development of TLR3 activation-based immune treatment for people with HIV infection.

## Ethics Statement

In this *in vitro* study, we obtained primary human monocytes from the Immunology Core at the University of Pennsylvania School of Medicine. The Core has the Institutional Review Board approval for blood collection from healthy donors. Anyone who obtains human cells from the Core is considered as secondary use of de-identified human specimens, which does not subject to human subject review by both NIH and IRB.

## Author Contributions

LG, LZ, XW, J-LL, and W-ZH designed the study. LG, X-QX, R-HZ, J-BL, BZ, and HL performed the experiments. W-ZH supplied reagents needed for this study. LG analyzed and interpreted the data and wrote the manuscript. LG and W-ZH reviewed and revised the manuscript. All the authors have read, reviewed, and edited the manuscript and agreed for submission to this journal.

## Conflict of Interest Statement

The authors declare that the research was conducted in the absence of any commercial or financial relationships that could be construed as a potential conflict of interest.
