# Supplementary material for: Human Intestinal Epithelial Cells Release Antiviral Factors That Inhibit HIV Infection of Macrophages
Source: Front Immunol. 2018 Feb 19;9:247. doi: 10.3389/fimmu.2018.00247 (PMC5825896; doi:10.3389/fimmu.2018.00247)
Supplement: Supplementary file 1 [file data_sheet_1.PDF]

Supplementary Material

Supplementary Figures：

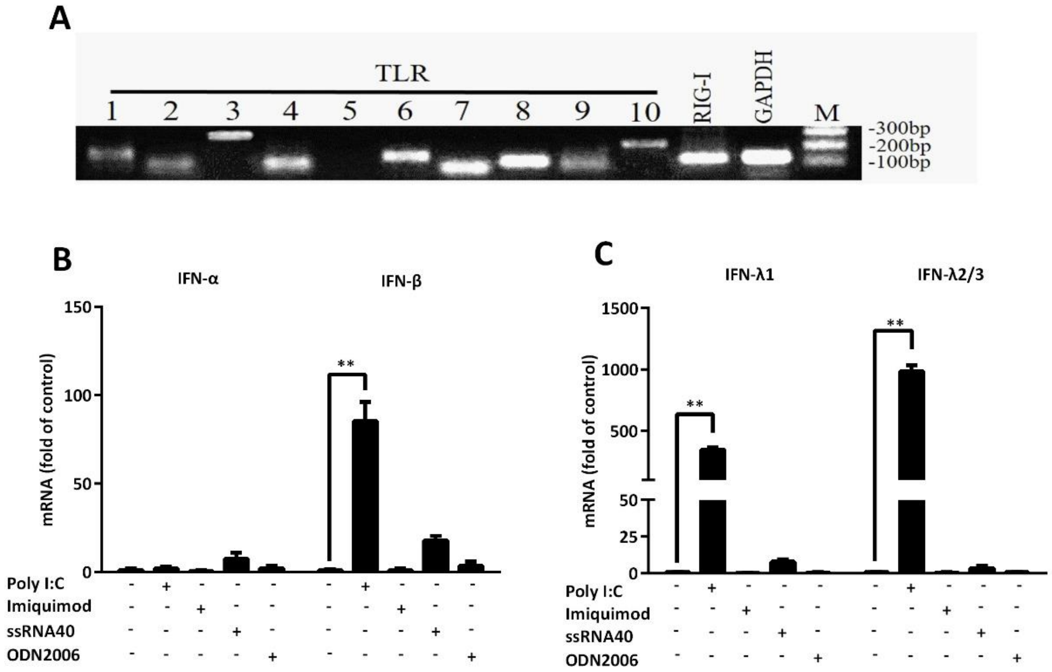

Supplemental Figure 1. Effect of TLRs activation on IFN expression. (A) Expression of TLRs in IECs. Total cellular RNA was subjected to the qRT–PCR with the primers specific for toll-like receptors (TLRs) 1–10 and RIG-I. Amplified PCR products were displayed on 2% agarose gel. (B) IECs were transfected with poly I:C (10μg/ml), Imiquimod (10μg/ml), ssRNA40 (10μg/ml) or ODN2006 (5μM) for 12h. Total cellular RNA was subjected to the qRT–PCR for the mRNA levels of IFN-β and IFN-λ. The results are the mean±SD of triplicate cultures, representative of three independent experiments (*P＜0.05, **P＜0.01).

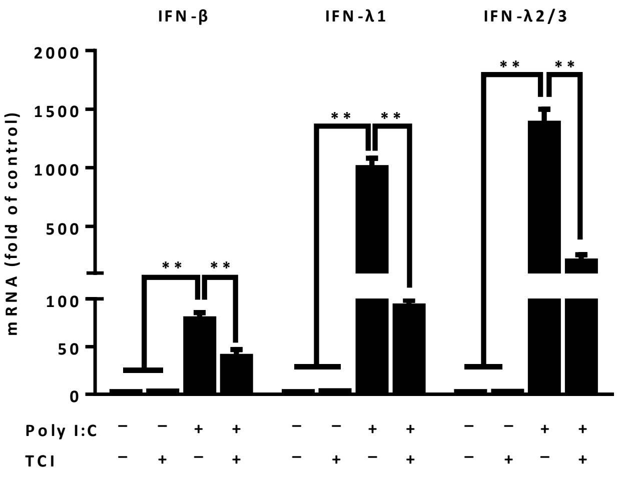

Supplemental Figure 2. Role of TLR3 in Poly I:C-mediated IFN induction. IECs were pretreated with 100nM TCI for 1h and then stimulated with 1μg/ml Poly I:C for 12h.

Total cellular RNA was subjected to the qRT-PCR for the mRNA levels of IFNs. Asterisks indicate that the differences between the indicated groups are statistically significant (*P＜0.05, **P＜0.01). TCI: TLR3/dsRNA complex inhibitor.

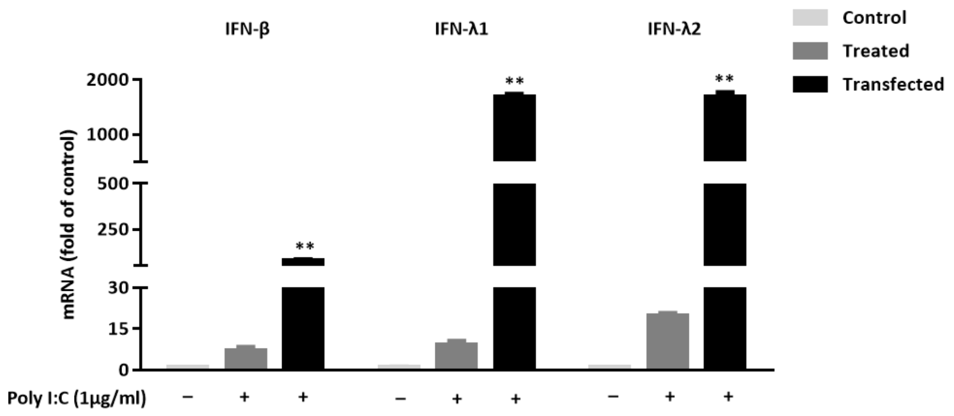

Supplemental Figure 3. Effect of TLR3 activation on IFN-β and IFN-λ. IECs were directly added or transfected with poly I:C (1μg/ml) for 12h. Total cellular RNA was subjected to the qRT-PCR for the mRNA levels of IFN-β and IFN-λ. The results are the mean±SD of triplicate cultures, representative of three independent experiments (*P＜0.05, **P＜0.01).
